# Staged endoscopic submucosal dissection for a giant gastric fundus lesion: a strategy for the incompletable

**DOI:** 10.1055/a-2719-8232

**Published:** 2025-11-14

**Authors:** Guanyi Liu, Chong Liu, Xudong Zhao, Long Rong

**Affiliations:** 1 26447 Endoscopy Center, Peking University First Hospital, Beijing, China

A 73-year-old female presented with anemia and underwent gastroscopy at a local hospital, which identified a large villous lesion in the gastric fundus measuring approximately 7 cm × 6 cm ( Fig. 1 ). The lesion was attached to the gastric fundus and cardia via a pedicle-like structure. Biopsy confirmed high-grade intraepithelial neoplasia (HGIN). Given multiple comorbidities, the patient was considered high-risk for surgery, and ESD was elected ( Video 1 ).

**Fig. 1:**
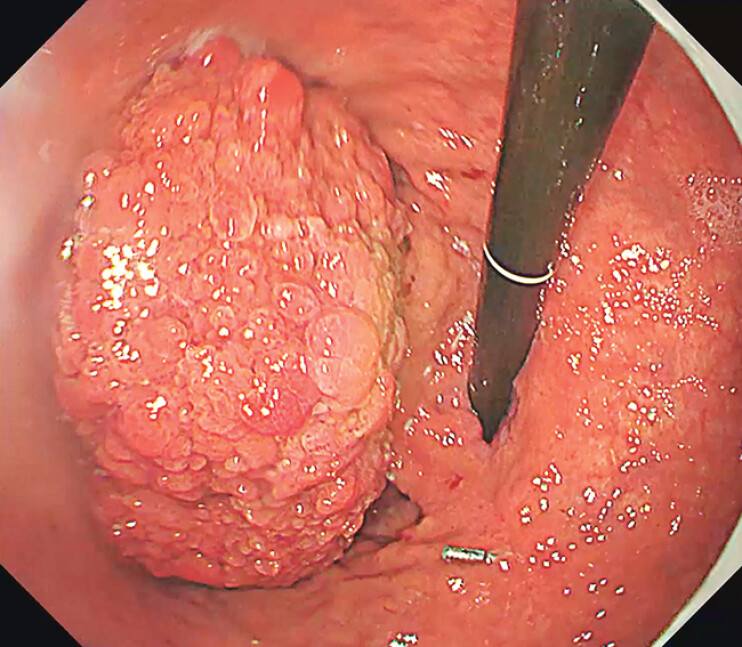
A large villous lesion was located in the gastric fundus, measuring approximately 7 cm × 6 cm. The lesion was attached to the gastric fundus and cardia via a pedicle-like structure.

Staged ESD for a large villous lesion in the gastric fundus.Video 1

Due to the considerable size of the lesion, visual exposure of the submucosal layer was inadequate. Traction assistance was provided sequentially using a clip-with-rubber-band system and a snare. Numerous large vessels within the lesion were identified and managed with clips and hemostatic forceps coagulation ( Fig. 2 ). The initial ESD session lasted 7.5 hours. Although most of the lesion was resected, the remaining portion – which still contained multiple large vessels – was endoscopically inaccessible despite repeated attempts. The procedure was therefore discontinued.

**Fig. 2:**
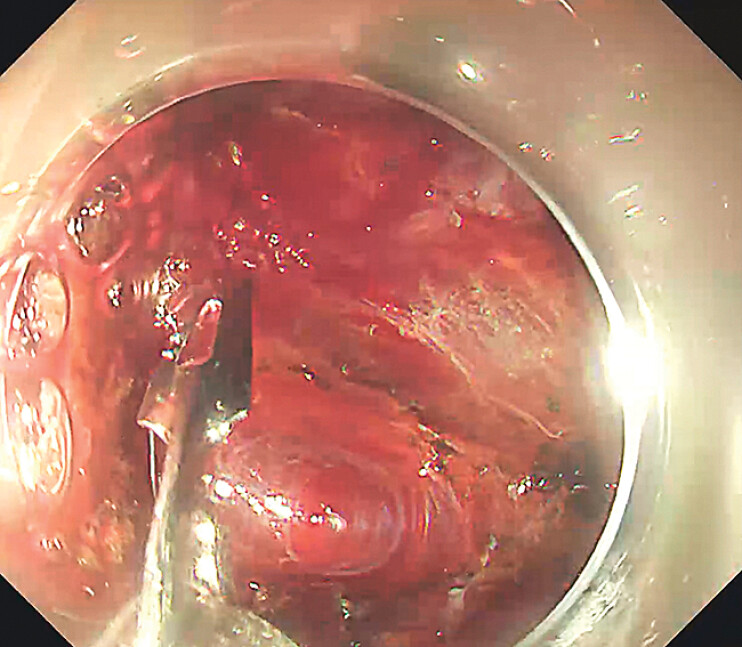
Numerous large vessels within the lesion were identified and managed with clips and hemostatic forceps coagulation.

Postoperatively, the patient received proton pump inhibitor therapy, prophylactic antibiotics, and nutritional support. Repeat gastroscopy 1 week later revealed extensive necrosis ( Fig. 3 ). The necrotic tissue was retrieved with a snare. The residual lesion had become clearly demarcated and readily accessible, enabling completion of resection via endoscopic piecemeal mucosal resection (EPMR) ( Fig. 4 ).

**Fig. 3:**
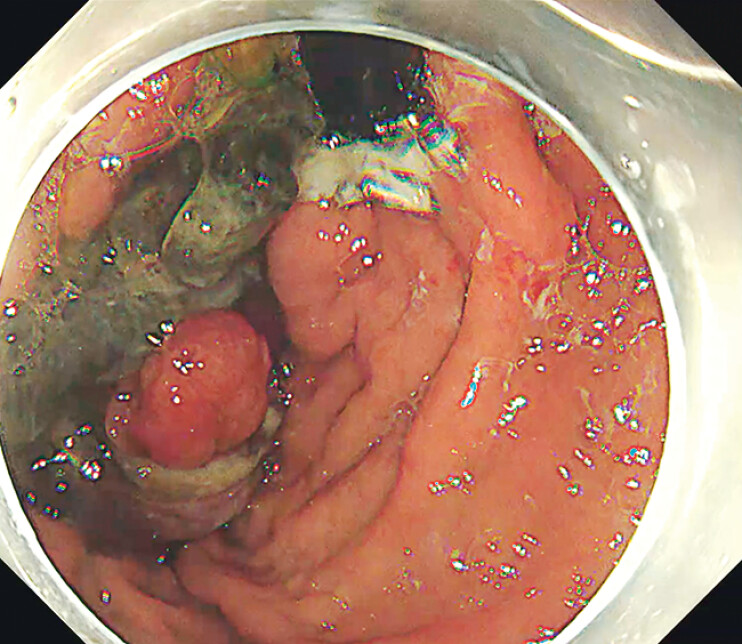
Repeat gastroscopy one week later revealed extensive necrosis.

**Fig. 4:**
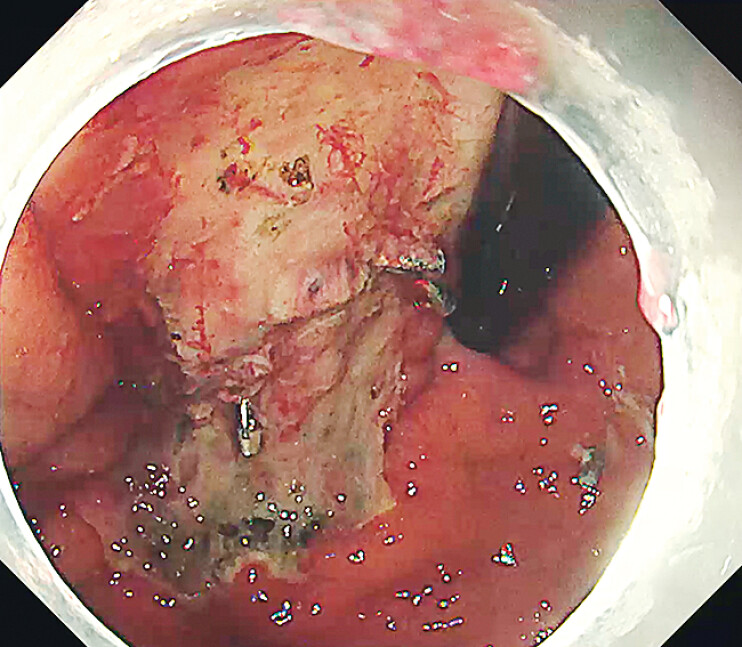
The residual lesion was completely resected via EPMR.

The patient recovered uneventfully and was discharged. Final pathology reported a pyloric gland adenoma with low-grade and focal HGIN. A 3-month follow-up endoscopy showed complete healing without residual or recurrence ( Fig. 5 ).

**Fig. 5:**
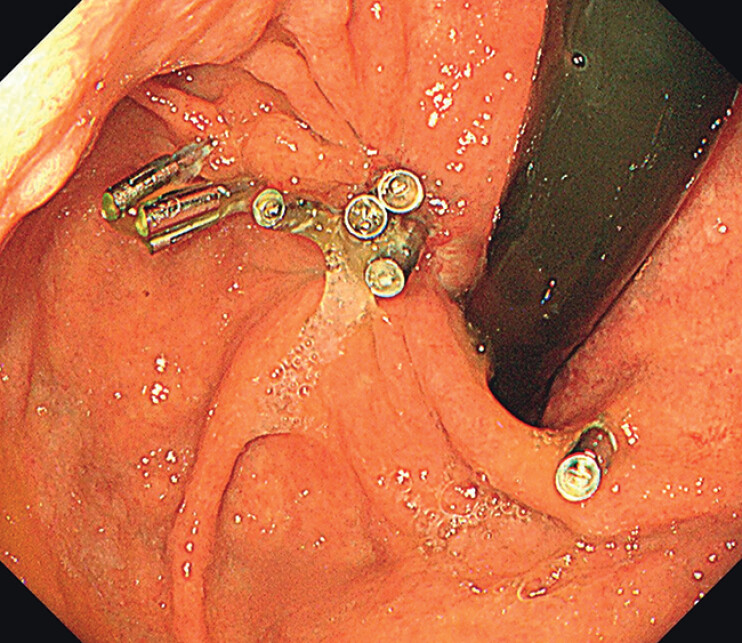
A 3-month follow-up endoscopy showed complete healing without residual or recurrence.

Staged ESD has previously been described by Japanese researchers for the management of circumferential Barrettʼs carcinoma 1 . To our knowledge, this is the first reported application of this strategy for a giant gastric lesion. We demonstrated a novel, safe endoscopic option for complex gastric lesions, expanding treatment possibilities for high-risk patients and highlighting techniques for managing challenging lesions.

Endoscopy_UCTN_Code_TTT_1AO_2AC

## Notes

Endoscopy E-Videos https://eref.thieme.de/e-videos
*E-Videos* is an open access online section of the journal *Endoscopy* , reporting on interesting cases and new techniques in gastroenterological endoscopy. All papers include a high-quality video and are published with a Creative Commons CC-BY license. Endoscopy E-Videos qualify for HINARI discounts and waivers and eligibility is automatically checked during the submission process. We grant 100% waivers to articles whose corresponding authors are based in Group A countries and 50% waivers to those who are based in Group B countries as classified by Research4Life (see: https://www.research4life.org/access/eligibility/ ). This section has its own submission website at https://mc.manuscriptcentral.com/e-videos .
